# Improved drug safety through intensive pharmacovigilance in hospitalized pediatric patients

**DOI:** 10.1186/s40360-017-0186-x

**Published:** 2017-12-08

**Authors:** Alan O. Vázquez-Alvarez, Lorena Michele Brennan-Bourdon, Ana Rosa Rincón-Sánchez, María Cristina Islas-Carbajal, Selene G. Huerta-Olvera

**Affiliations:** 1 Instituto de Terapéutica Experimental y Clínica (INTEC). Departamento de Fisiología. Centro Universitario de Ciencias de la Salud, Universidad de Guadalajara, Guadalajara, Jalisco Mexico; 2 Comisión para la Protección contra Riesgos Sanitarios del Estado de Jalisco (COPRISJAL), Guadalajara, Jalisco Mexico; 3 Departamento de Biología Molecular y Genómica. Centro Universitario de Ciencias de la Salud, Universidad de Guadalajara, Guadalajara, Jalisco Mexico; 4 Departamento de Ciencias Médicas y de la Vida. Centro Universitario de la Ciénega, Universidad de Guadalajara, C.P. 47820 Guadalajara, Jalisco Mexico

**Keywords:** Adverse drug reaction, Pharmacovigilance, Intensive pharmacovigilance, Drug safety

## Abstract

**Background:**

The aim of this study was to detect and analyze Adverse Drug Reactions (ADRs) through Intensive Pharmacovigilance (IPV) in hospitalized pediatric patients to improve drug safety.

**Methods:**

A prospective 6-month cross-sectional study was performed in the pediatric service of a regional hospital in Mexico in order to assess hospitalized children from 1 day to 18 years old. The inclusion criteria were: both genders, all hospitalization causes, and at least one prescribed medication (indistinct drug group). Notifications were performed through medical visits, phone calls, or spontaneous reports. ADR suspicions were assessed with severity scales: Naranjo algorithm, Schumock & Thornton and Hartwig and Siegel.

**Results:**

From a total of 1083 hospital admissions, 19 ADRs were recorded. The average age of patients in years was 7.2 (±5.9). The causality assessment in this study showed that most of the ADRs were probable (68.4%) and 4 certain (8.2%); causality was mainly attributed to antibiotics (AB) and an antiepileptic drug. We found a relationship of AB with ADRs (*p* < 0.05) with an increased risk at the third day of prescription (*p* < 0.05). The average severity was level 2 and 21% were classified as “preventable”. Lastly, an increase in hospital stay associated with ADRs (*p* < 0.05) and with concomitant medications (*p* < 0.05), was also found. The most severe ADRs were hemolysis and toxic epidermal necrolysis.

**Conclusions:**

IPV was an effective tool for ADR prevention, detection, and treatment in hospitalized patients. The intensive monitoring approach in pharmacovigilance amplifies ADR detection and this translates into the improvement of drug safety in children.

## Background

Adverse Drug Reactions (ADRs) are defined by the World Health Organization as “Any noxious, unintended and undesired effect of a drug which occurs at the dosages used in humans for prophylaxis, diagnosis or therapy" [1–3]. Globally, the presence of ADRs has increased, showing an incidence of 2.2 million in 1994 [4, 5] and 10 million in 2014 [6, 7]. In addition, the prevalence of hospital admissions for Drug-Related Problems (DRPs) has reached up to 28% in the US and the annual cost for this cause is estimated in 170 billion US dollars [8].

The pediatric population is one of the most vulnerable groups to ADRs [9]. The WHO Global Individual Case Safety Report (ICSR) database (VigiBase®), reported rates of ADRs in 7.7% in children from 0 to 17 years [10]. However, these reports seem to show underestimated rates as other studies with a higher incidence of ADRs reaching >7000 serious or fatal ADR reports in children, mainly ≤2 years old, have been reported [9, 11, 12]. This susceptibility is due to different factors such as physiological immaturity, which determines changes in pharmacokinetic parameters. As a result, in the pharmacological response, dose modifications in pediatric patients should be calculated based on weight, body surface area, gestational age, as well as liver and kidney function, among others. Moreover, there is limited scientific evidence on the effectiveness and drug safety in this population since the standardization of dosage strategies of many drugs is extrapolated from adults, and as a result, children are considered therapeutic orphans [11, 13–17].

There is a need to propose valuable methods that can detect ADRs early in the pediatric population [17]. In order to reduce the global occurrence of ADRs in hospitals, some strategies have been implemented with the primary objective of diminishing ADR incidence or reducing inpatient costs, such as computerized systems, coded administrations, as well as computerized physician order entries, and clinical decision support systems, in spite of the spontaneous reporting of possible drug caused adverse events [11, 18]. While spontaneous reporting underestimates the incidence of ADRs and the use of computerized systems for monitoring provides the best results, there is no single best method; however, the use of multiple strategies maximizes the quantification of ADRs [19].

ADRs represent a significant health problem resulting in altered therapeutic strategies, increased hospital stay, as well as higher morbidity and mortality rates, and elevated hospital costs. Intensive pharmacovigilance (IPV) is the systematic monitoring of the occurrence of adverse events resulting from drug use during the entire length of prescription [1, 3, 20] and is considered a useful tool to prevent, identify, and treat preventable and non-preventable adverse reactions to medications. Furthermore, pharmacovigilance activities in the pediatric population have demonstrated to favor the assessment of drug safety [9, 20]. However, in order to improve ADR detection, these activities need to be promoted in the hospital pediatric services. In Mexico, there is no specific data about ADR incidence in the pediatric population. Also, studies addressing ADR monitoring activities such as IPV, are scarce in Mexico, especially related to hospitalized pediatric patients. The purpose of the study was to detect and evaluate the ADRs in hospitalized children of a regional hospital in Western Mexico by the IPV method in order to improve medication safety.

## Methods

The aim of this study was to detect and analyze Adverse Drug Reactions (ADRs) through Intensive Pharmacovigilance (IPV) in hospitalized pediatric patients to improve drug safety. A prospective cross-sectional pharmacovigilance study was conducted in the pediatric service of a regional hospital in Mexico for 6 months. The emergency, intensive care, and oncology departments were excluded. Inclusion criteria were patients from 1 day to 18 years old from pediatric hospitalization floors 3 and 4 of the New Hospital Civil of Guadalajara “Dr. Juan I. Menchaca” Jalisco, Mexico. This study was classified as “no risk” [21], so only verbal consent was required from the parent or legal guardian of the child in order to participate in the study. Approval was obtained from the Research Ethics Committee of the New Hospital Civil of Guadalajara “Dr. Juan I. Menchaca” of the New Hospital Civil of Guadalajara “Dr. Juan I. Menchaca”, with the registration number: 1225–12. For those patients who refused to participate in this study, they were still subject to the corresponding evaluations and treatments before any ADR suspicion.

During the evaluation period, gender, and reason for admission were open with at least one prescribed medication (indistinct drug group) during the hospital stay. Informed verbal consent for suspecting ADRs and the implementation of relevant tools were obtained. Exclusion criteria were: no prescription medication present during the hospital stay, declined verbal consent for suspected ADRs, or if the patient or caregiver did not answer the questions at the time of the interview to detect ADRs.

Initially, the ADR evaluator was presented to the medical team of the pediatric service (attending physician, medical resident, intern, nurse, and head nurse). Every 24 h, a visit with each patient was performed. For new admissions, information was provided (including education and suspicions of ADRs). Patients were told to keep in touch with the attending medical personnel or by the evaluator in case of any suspected ADRs. For the identified cases, an assessment of suspected cases was performed by examination and review of the medical and nursing records. In the case of suspecting ADRs, we proceeded to collect information and patients were invited to participate in the study. Once the patient/caregiver/family member gave their verbal consent for this study, we proceeded to conduct a review of the medical records to determine age, sex, diagnosis, and the characteristics of the prescribed treatment (implicated drugs, polypharmacy [≥3 drugs], indication, day and dose), affected organs or systems as well as the drug reaction (including severity and progression). Drug-drug interaction analyses were performed and possible medication errors were evaluated (supra and infra-dose therapy, infusion rate, inadequate route of administration, etc.). Once these were discarded, Naranjo algorithm was used to determine causality [22, 23].

To assess the severity and predictability of ADRs, the Hartwig and Siegel classification [24] and the Schumock and Thornton questionnaire [25] were used respectively, to evaluate the adverse events through a series of questions. In the case of suspected ADRs the official format for suspected ADRs issued by the Comisión Federal para la Protección contra Riesgos Sanitarios - COFEPRIS (*Federal Commission for the Protection against Sanitary Risks)* was completed. Once the report was finalized, it was handed out to the pharmacist responsible of the hospital and turned to the Hospital Pharmacy team for its evaluation and the corresponding internal registration.

To describe the drugs involved in this study, the Anatomical Therapeutic Chemical (ATC) Classification by the WHO, [26] and for affected organs and systems, the System Organ Class (SOC) Classification, proposed by the Uppsala Monitoring Center [27], were used. The following variables were calculated: 1) ADR frequency (based on the total number of hospitalized children within the study period); 2) ADR incidence (ADRs observed in children in the total hospital length of stay in days during the study period × 1000); 3)Percentage of severity (calculated as the level of severity in all ADRs, starting at level 3 Hartwig and Siegel × 100) and 4) Percentage of preventable ADRs (all ADRs reported as “preventable” by the algorithm Schumock × 100) [14]. Other results were analyzed using descriptive statistics, mean as a measure of central tendency and standard deviation as a measure of dispersion for quantitative data; qualitative data were expressed in absolute frequencies, percentages, and ratios.

Results are expressed as averages and percentages. The data were analyzed using Chi-square test or U Mann-Whitney test as needed and a *p* value <0.05 was considered significant.

## Results

A total of 1083 hospital admissions were recorded during the 6-month period. Study group characteristics are described in Table 1. The male: female ratio was 1.3:1. Registered patients were classified into two groups whether they were younger or older than 1 year old. The mean age (± SD) observed for all patients was 4.3 (± 0.52) years. A total of 1517 diagnoses were recorded during the study period and the most representative groups were respiratory (457; 30%) and neurological (161; 11%), including one obstetrics and gynecology case of asymmetric intrauterine growth restriction.

**Table 1:** Pediatric population distribution by age group (<1 year and ≥1 year)

Variables	<1 year (age in months)	≥1 year (age in years)	General [mean(±SD)]
Age	0.72 (±0.52)	14.0 (±10)	4.3 (±0.5)
Gender (male/female)	281/161	343/298	624/459
Weight (kg)	11.6 (±7.0)	49.4 (±35.8)	61.0 (±42.8)
Hospital stay (days)	11 (+19)	8 (+12)	9 (+14)
Diagnostic Group			
-Respiratory	272	185	457
-Neurology	42	119	161
-Blood and Hematopoietic	67	75	142
-Gastrointestinal	46	86	132
-Genito-urinary	27	105	132
-Infectious Disease	62	39	101
-Development and Nutrition	36	24	60
-Surgery	17	39	56
-Legal-Medical	19	18	37
-Dermatology	7	29	36
-Soft tissues	7	29	36
-Metabolic	12	20	32
-Genetic	18	14	32
-Head and neck	12	16	28
-Trauma and Orthopedics	7	16	23
-Cardiovascular	6	10	16
-Autoimmune	/	18	18
-Oncology	/	14	14
-Toxicology	/	3	3
-Obstetrics and Gynecology	1	/	1

Pediatric population distributed by age group (<1 year and ≥1 year). The distribution of the pediatric population (male and female patients) is shown by age group, in patients with less than one year of age and those aged one year or older

**Fig. 1:**
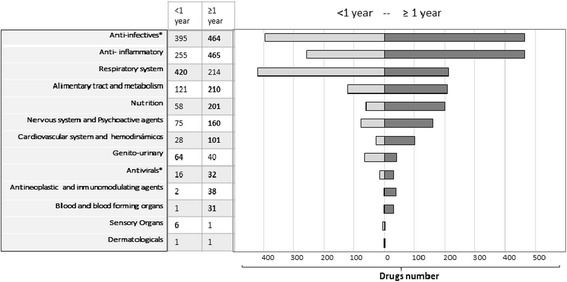
Pharmacological drug distribution by age groups *In the ATC classification the antibiotics and antivirals group are together, however in this figure they are placed separately in order to observe patients of each group individually

The drug delivery groups according to the ATC code are described in Fig. 1. The most common prescribed drug classes were antibiotics [AB] (29.4%) and anti-inflammatory drugs (21%). A total of 19 ADRs were recorded, 18 children developed just one ADR during the hospital stay and one presented two ADRs with different time periods during the study evaluation (Table 2). The overall estimated incidence of ADRs in children was 17 per 1000 children. The mean age was 7.2 years (± 5.9) with a female predominance (63%). The incidence of ADRs in days was 1.8 per 1000 children days. The average hospital stay (without ADRs) was 9 (+14) days. Lastly, the average for concomitant medications was 3.7 (± 2.7) and a significant association with the risk of ADRs (*p* < 0.05; Chi -square), was found.

**Table 2:** Characteristics and classification of ADRs

Variables	General [mean(±SD)]
Age (years)	7.2 (±5.9)
Number	19
Concomitant medications	3.7(±2.7)*
Hospital stay (days)	14 (+17)+
Naranjo [average points]	6.2 (±2)
1–4 points (Possible)	2
5–8 points (Probable)	13
≥ 9 points (Certain)	4
Schumock & Thornton Scale	
Preventable	4/19 (21%)
Not preventable	15/19 (79%)
Hartwig & Siegel [average level]	2.3 (±1.0)
Level 1	4
Level 2	8
Level 3	5
Level 4	1
Level 5	1

Classification of ADRs ADRs were classified according to the Naranjo, Schumock & Thornton and Hartwig & Siegel scale

*The comparison between ADR group and without-ADR was significant (*P* < 0.05; Chi-square)

+The comparison of hospital stay between ADR group and without-ADR was significant (*P* < 0.05; U Mann-Whitney)

*ADRs* Adverse Drug Reactions

Considering AB as one of the most prescribed drug groups, we found a relationship between the number of AB prescribed with the ADRs reported. There was a high incidence of ADRs caused by antibiotics (*p* < 0.05; Chi-square) Then, we evaluated the relationship between the occurrence of ADRs and the first, second, third, fourth, and fifth day of AB prescription and we only observed a significant difference in the third day of AB prescription (*p* < 0.05; Chi-square).

Based on the causality determined by the Naranjo algorithm, we observed 2 cases as “possible”, 13 patients as “probable” (68.4%) and 4 cases as “certain” (21%). These 4 cases were attributed to three antibiotics (amoxicillin, amikacin, and penicillin) and the anticonvulsant carbamazepine. The predictability of ADRs, determined by Schumock and Thornton scale was 20%. Hartwig and Siegel severity scale was predominantly in Level 2 (8 cases; 42%). In other words, discontinuation of the drug was required without the administration of an antidote, medicine or an increased length of hospital stay, with an average of 2.3 (± 1) days. The average stay of patients with ADRs in days was 14 (+17) and the percentage of total severity was 36.8%. Furthermore, we found a significant increase in hospital stay compared to the average hospital stay (*p* < 0.05; U Mann-Whitney). The more involved drug groups, according to the ATC code, included anti-infective (63%) and nervous system (15.7%) medications. Distribution of ADRs according to organs and systems was mainly skin and annexes (11 cases) characterized by rash and severe itching, followed by the nervous system (5 cases) portrayed by anxiety, headache, and drowsiness (Table 3). Nevertheless, we did observe the appearance of diplopia (by carbamazepine), paresthesia (by diphenidol), and anaphylaxis (by metronidazole). These reactions did not require medical intervention. The most severe ADRs were hemolysis (1 case) and toxic epidermal necrolysis (classified as skin and annexes, 1 case). It is important to mention that for these two most severe reactions, patients were given continuous monitoring during their hospital stay until discharge, both without any consequences. We synthesize the most relevant findings when comparing the pediatric population “with ADRs” against those “without ADRs” in Table 4.

**Table 3:** Therapeutic groups and affected organs related to ADRs

*n* = 19 ADRs		Total
Pharmacological groups (ATC code-First level)		
-Antiinfectives (J)		12**
-Nervous System (N)		3
-Blood and blood forming organs (B)		1
-Sensory organs (V)		1
-Alimentary tract and metabolism (A)		2
Distribution by affected organs and systems		
-Nervous system	Anxiety (2), headache(2), drowsiness (1)	5
-Skin and annexes	Rash (9), intense pruritus (2)	11
-Blood and blood forming organs	Hemolysis (1)	1
-Cardiovascular system		
-Immunological system	Hypertension (2), hypotension (1)	3
-Gastrointestinal tract	Fever (2), anaphylaxis (1)	3
-Sensory organs	Diahrrea (1)	1
-Muscle-skeletal system	Diplopia (1)	1
-General effects	Paresthesias (1)	1
	General discomfort (1)	1

Therapeutic groups and affected organs related to ADRs ADRs were classified according to the ATC Code-First Level and their distribution by affected organs and systems

**The relationship between the amount of AB and ADR was significant (*s* < 0.05; Chi-square) and also the presence of ADR and third day of AB (*P* < 0.05; Chi-square)

*ADRs* Adverse Drug Reactions

**Table 4:** Comparison between pediatric patients with and without ADRs

Variable	Without ADRs *n* = 1065 [mean(±SD)]	With ADRs *n* = 18 [mean(±SD)]	*P* value
Age (years)	4.3 (±0.52)	7.2 (±5.9)	NS
Hospital stay (days)	9 (+14)	14 (+17)	0.008
Concomitant medications	2.3 (±1.95)	3.7(±2.7)	0.001
Number of prescribed AB	0.78 (±0.03)	1.3 (±0.40)	0.001
Relationship with day of AB administration and ADR risk:			
1 day			NS
2 days			NS
3 days			0.010
4 days			NS
5 days			NS
6 days			NS

Comparison between ADRs and without ADRs population We show some of the most important variables analyzed among the study groups

*ADRs* Adverse Drug Reactions

## Discussion

A total of 19 ADRs were reported with an incidence of 1.7% in relation to hospital admissions. In contrast, Arulmani et al. [28] found in the pediatric group, an ADR incidence of 11.6%. Meanwhile, Telechea et al. [14] found an incidence of 19.5% in the pediatric intensive care unit. These differences in ADR incidence compared to other studies could be attributed to ethnic, genetic, and dietary factors. Others factors are the disease pattern, socioeconomic status, healthcare infrastructure, and the detection method employed [29]. The IPV monitoring of ADRs in our study, unlike the study by Arulmani et al., was able to discard those suspicions caused by DRPs, an advantage the spontaneous report does not possess when the information lacks an in-depth analysis. Furthermore, the high incidence in the Telechea et al. study may be due to the small group studied in comparison to our study group.

The drug group with the largest number of ADRs was AB and 75% of these were classified as “certain”. This finding is consistent with studies reported by Arulmani [28], Murphy [30] and Suh [31], even though the percentage caused by AB was higher in our group than those reported by others. For example, Hernández et al. [32] demonstrated that 38% of ADRs were caused by AB in a study conducted in the IMSS (*Mexican Institute of Social Security)*. Similarly, in the review by Ponte [33], 26.1% of ADRs were attributed to antibiotics, surpassed only by cardiovascular drugs, which were absent in this study. Moreover, a significant incidence of ADRs caused by antibiotics and their relationship with the third day of prescription found in our study, highlights the importance that must be given in the surveillance of these drugs, particularly in pediatric patients.

Although the average severity of ADRs was “level 2”, which establishes: “… *no increase in the length of hospital stay*”, there was an increased tendency in our study to favor the length of hospital stay in patients with ADRs compared to the average of all hospitalized patients. This increase could be due to factors related to the event (monitoring, treatment changes or related effects), which conditioned modifications in the original treatment plan or prognosis. Furthermore, an increased length of stay may have an effect on the hospital’s economy, described by P. Hernandez [32] as “dollar for dollar”, generating an increase of those unscheduled resources in order to handle ADR suspicions. The additional medications used to treat the ADRs in developing countries such as Mexico or Ethiopia, [34] increase the cost of each treatment. As reported by Hernández.

Polypharmacy observed in this study included 3.7 (± 2.7) concomitant medications, potentially leading to increased risk of interactions or ADRs. Many studies have shown that polypharmacy is an important risk for drug-drug interactions and ADRs [17, 35, 36]. In our study we confirm that the additive risk caused by the significant increase of ADRs with ≥3 drugs, especially with AB (*p* < 0.05), could be a predictor of ADRs.

The Naranjo algorithm is endorsed internationally as a tool for causality of ADRs. However, it has limitations that hinder the clarification of suspicions and involve ethical implications. For example, it is necessary to perform placebo administration (which may be questioned by the patient’s parent/guardian) or the re-administration of the suspected drug when the severity of the reaction is significant (hemolysis, etc.). As a result, a lower causality than expected is established. However, in most pediatric studies, the Naranjo Algorithm is preferred due to its simplicity. Nonetheless, the validity and reliability of this tool has been demonstrated in adults but not in the pediatric population [9, 35].

The pediatric population is one of the most vulnerable groups to present ADRs. Aagaard et al. in their review [15] found that >40% of ADRs are presented in patients aged 1–10 years and in our study we observed 79% of ADRs in this age group of 1–10 years. This increased tendency of ADRs could be attributed to admission diagnoses combined with an increased use of AB, concomitant medications, and an increased hospital stay. In addition, in the age group of <1 year (less than one year), we observed an increased susceptibility to diseases of the respiratory system, urinary system, and sensory organs (Table 1). This increased susceptibility could be the result of their immature immune system. However, further studies are required to clarify the increased rates of ADRs in patients of this age group.

The most affected organs or systems were skin and annexes, as well as the central nervous system (Table 3). Our findings are consistent with several studies where a high percentage of clinical manifestations were related to these systems [15, 28, 37].

The most important challenge encountered during the development of the study, was the lack of professional culture in ADR reporting, including the lack of suspicion when a suspected ADR was present, in addition to the false belief that there are “expected” effects as well as the lack of knowledge in ADR reporting and analysis. These limitations are similar to those described by John et al. [38], emphasizing the importance of strengthening the education of health personnel in clinical training of ADR reporting. Our study was conducted in a teaching hospital of Western Mexico where there are periodic rotations of the health team, so education in reporting ADR suspicions was continuously provided. During this process, we encountered some limitations of this study, described as “Inman’s seven deadly sins” [39] characterized by: fear, indifference, greed, guilt, complacency, ignorance, and timidity.

It is a fact that Pharmacovigilance will eventually develop a secure and coherent utilization of medications [17]. Furthermore, the implementation of IPV increased the quality of the attention and showed an improvement in the evaluation of drug-related safety by the health care team, which was reflected in the overall enhanced patient care. Reasonably, this increased attention is equally reflected as an increase in the occurrence of suspected ADRs and other DRPs, which can be explained by Muehlberger et al. [ 40], where the monitoring of adverse drug reactions provided a higher incidence value in comparison with spontaneous reports. As a result, we have confirmed that pharmacovigilance monitoring of ADRs improved the evaluation and understanding of the drug-related safety issues in our study group [9]. There are several limitations that must be considered in terms of interpreting the findings of the study. For example, the short implementation period and the use of unlicensed or off-label medications in children was not considered as a potential risk factor in the analysis; another important limitation is the potential selection bias of those patients who did not provide their consent for the study and were not included in the analysis, and lastly, the study was only conducted in one hospital and one service area.

## Conclusion

We found that IPV in hospitalized pediatric patients allowed a careful observation of patients during their hospital stay, as well as an increased detection of DRPs and suspected ADRs. As a result, we were able to establish the frequency and type of drugs more related to ADRs and thus, detect and prevent ADRs through IPV with a direct impact on pediatric drug safety.

## Abbreviations

ADRs: Adverse Drug Reactions
ATC: Anatomical Therapeutic Chemical
AB: Antibiotics
COFEPRIS: Comisión Federal para la Protección contra Riesgos Sanitarios
DRPs: Drug-related problems
ICSR: Individual Case Safety Report
IMSS: Mexican Institute of Social Security
IPV: Intensive pharmacovigilance
SD: Standard Deviation
SOC: System Organ Class
